# Assessment of Toxicity of Some Penta- and Hexacoordinated Organotin(IV) and Tetracoordinated Tin(II) Complexes of Heterocyclic *β*-Diketones

**DOI:** 10.1155/BCA/2006/60140

**Published:** 2006-05-23

**Authors:** Asha Jain, Sanjiv Saxena, Audhesh K. Rai, Prabhu N. Saxena

**Affiliations:** ^1^Department of Chemistry, University of Rajasthan, Jaipur 302004, India; ^2^Department of Zoology, Toxicology Laboratory, School of Life Sciences, Dr. B.R. Ambedkar University, Agra 282004, India

## Abstract

A number of penta- and hexacoordinated organotin(IV) complexes and tetracoordinated tin(II) complexes of compositions ${\text{Me}}_{2}\text{SnCl[RCO}\overset{⎴}{{\text{C:CON(C}}_{6}{\text{H}}_{5}\text{)N:C}}{\text{CH}}_{3}\text{]}$ (where R = − CH_3_, −p−ClC_6_H_4_, and −C_6_H_5_), ${\text{Me}}_{2}\text{Sn[RCO}\overset{⎴}{{\text{C:CON(C}}_{6}{\text{H}}_{5}\text{)N:C}}{\text{CH}}_{3}{\text{]}}_{2}$ (where R = −CH_3_, and −C_6_H_5_), and Sn(II) ${\text{[RCO}\overset{⎴}{{\text{C:CON(C}}_{6}{\text{H}}_{5}\text{)N:C}}{\text{CH}}_{3}\text{]}}_{2}$ (where R = −p−ClC_6_H_4_ and −C_6_H_5_) were screened for their toxicity against *Musca domestica* (house fly). In general, organotin(IV) complexes contribute more to the activity than tin(II) complexes.

## INTRODUCTION

There has been considerable interest in the chemistry of penta- and hexacoordinated organotin(IV) complexes derived from various organic ligands due to their structural and stereochemical aspects. In marked contrast to the well-documented chemistry of organotin(IV) complexes [1–3], the number of reports available in the literature on the corresponding tin(II) [4, 5] complexes are rather scanty. Organotin(IV) complexes are put to use in various fields [6–8] and exhibit potential biological applications [9–11] such as insecticidal, fungicidal, and antitumor activities.

In view of the interesting results obtained in our previous communication [12] dealing with the toxicity of organotin(IV) and tin(II) complexes of heterocyclic *β*-diketones, it was considered relevant to screen a series of penta- and hexacoordinated organotin(IV) complexes and tetracoordinated tin(II) complexes for their toxicity against *Musca domestica* (house fly). We now report the structure-activity relationship of some penta- and hexacoordinated organotin(IV) complexes and tetracoordinated tin(II) complexes of 4-acyl-2,4-dihydro-5-methyl-2-phenyl-3H-pyrazol-3-ones in detail.

## EXPERIMENTS

The organotin(IV) complexes and tin(II) complexes were prepared by the method advanced by Rai et al [13, 14]. These complexes were diluted with solvent to estimate the LC_50_ values for house flies (*Musca domestica*). The house flies (*Musca domestica*), 100 in each experimental set, were lightly anaesthetized with carbon dioxide and were acclimatised for 24 hours. at *circa*. 25°C in Fonda containers. The house flies were fed on milk soaked cotton pads. Each fly was then fully held with forceps and was treated with 1 *μ*L of preassigned dilution of the experimental compounds on the thorax. Controls (house flies) were treated with 1 *μ*L of acetone using an automated microapplicator. The so-treated flies were then returned to appropriately labelled containers, given access to milk-soaked cotton, and again maintained at * circa*. 25°C for 25 hours. during which mortality counts were made. The criterion for mortality was no response to probing; any movement was taken to indicate survival for the present investigation.

After approximate LC_50_ range was bracketed, a new stock solution of each experimental compound was serially diluted with acetone to obtain six concentrations (0.25%, 0.50%, 1.0%, 1.5%, 2.0%, and 4.0% by weight). Five replications per concentration were then tested. In each replication, the controlled flies were treated with 1 *μ*L of acetone. Post-treatment handling conditions were the same (see above).

The statistical analysis system (SAS) software package was used to estimate LC_50_ values and their fiducial limits (± standard error for each regression). Slopes of the probit regression obtained for populations of the house flies were then analyzed by the method of Steele and Torrie [15]. Present mortalities were corrected using Abbott's [16] formula.

## RESULTS AND DISCUSSION

The organotin(IV) and tin(II) complexes of compositions ${\text{Me}}_{2}\text{SnCl[RCO}\overset{⎴}{{\text{C:CON(C}}_{6}{\text{H}}_{5}\text{)N:C}}{\text{CH}}_{3}\text{]}$ (where R = −CH_3_, −p− ClC_6_H_4_, and −C_6_H_5_), ${\text{Me}}_{2}\text{Sn[RCO}\overset{⎴}{{\text{C:CON(C}}_{6}{\text{H}}_{5}\text{)N:C}}{\text{CH}}_{3}{\text{]}}_{2}$ (where R = −CH_3_ and −C_6_H_5_), and Sn(II) $\text{[RCO}\overset{⎴}{{\text{C:CON(C}}_{6}{\text{H}}_{5}\text{)N:C}}{\text{CH}}_{3}{\text{]}}_{2}$ (where R = −p−ClC_6_H_4_ and −C_6_H_5_) were synthesized, and the structures of these complexes have already been reported [13, 14] by us earlier on the basis of physicochemical and spectral (^1^H, ^13^C, and ^119^Sn NMR) evidences. The ligand (LH) employed for the synthesis of these complexes was prepared by a reported method [17], where LH=$\text{RCO}\overset{⎴}{{\text{C:C(OH)N(C}}_{6}{\text{H}}_{5}\text{)N:C}}{\text{CH}}_{3}$, R = −CH_3_ (AMPPOH), −p−ClC_6_H_4_ (CMPPOH), and −C_6_H_5_ (BMPPOH). These ligands (AMPPOH, CMPPOH, and BMPPOH) were found to be least active when tested against *Musca domestica* (house fly), but on complexation, the biological activity of the resulting complexes increased. The results of the screening of pentacoordinated and hexacoordinated organotin(IV) and tetracoordinated tin(II) complexes are listed in Table 1 and the complexes are arranged in descending order of biological activity.

**Figure 1:**
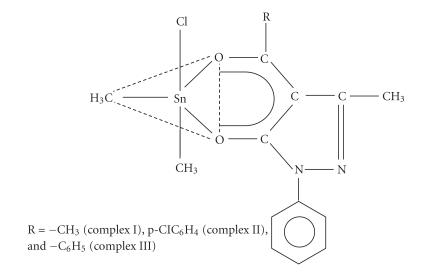
Monochloro(4-acyl-2,4-dihydro-5-methyl-2-phenyl-3H-pyrazol-3-onato) dimethyltin(IV).

In order to study the structure-activity relationship, some pentacoordinated diorganotin(IV) complexes of composition${\text{Me}}_{2}\text{SnCl[RCO}\overset{⎴}{{\text{C:CONC}}_{6}{\text{H}}_{5}\text{N:C}}{\text{CH}}_{3}\text{]}$ (where R = −CH_3_ (complex I), R = −p−ClC_6_H_4_ (complex II), and R = −C_6_H_5_ (complex III)) were synthesized and their structure was suggested previously [13] on the basis of spectral studies. ^119^Sn NMR spectral study reveals the presence of five coordinations around the central tin atom in these complexes (Figure 1).

In these complexes, chlorine atom is attached to the central tin atom. The presence of chlorine atom on organotin(IV) moiety is an important factor for imparting activity to the complexes I, II, and III. Further, among the dimethyltin chloro complexes, some substitutions were carried out on the ligand moiety. Complex I was more active than complexes II and III. Complex I has alkyl substitutent (R = −CH_3_) over the ligand; whereas complexes II and III have aryl substituents (R = −p−ClC_6_H_4_ and −C_6_H_5_). Further, complex II was more active than complex III because the former contains chlorine atom on the ligand moiety (R = −p−ClC_6_H_4_) while the latter lacks it (R = −C_6_H_5_).

**Figure 2:**
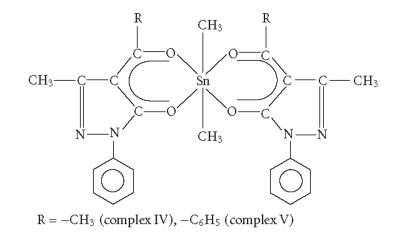
Bis(4-acyl-2,4-dihydro-5-methyl-2-phenyl-3H-pyrazol-3-onato) dimethyltin(IV).

In order to study the effect of coordination number, geometry around the central tin atom, delocalisation, and steric factors, some hexacoordinated organotin(IV) complexes of composition ${\text{Me}}_{2}\text{Sn[RCO}\overset{⎴}{{\text{C:CON(C}}_{6}{\text{H}}_{5}\text{)N:C}}{\text{CH}}_{3}{\text{]}}_{2}$ (where R = −CH_3_ (complex IV) and −C_6_H_5_ (complex V)) were synthesized. ^119^Sn NMR spectral studies [13] of the complexes IV and V reveal that these complexes possess distorted octahedral geometry around the central tin atom with methyl groups approximately trans to each other (Figure 2).

In these complexes, heterocyclic *β*-diketone behaves as bidentate ligand. The geometry of complex IV is confirmed [18] with the help of single crystal X-ray analysis which reveals C−Sn−C angle 162.1(3)°. Complex IV was more active than complex V. Complex IV possesses an alkyl substituent, while complex V possesses an aryl substituent over the ligand.

The pentacoordinated diorganotin(IV) complexes I, II, and III have a chlorine atom which is directly attached to the central tin atom which may be the plausible reason for enhanced activity of these complexes. The hexacoordinated complexes IV and V contain two heterocyclic *β*-diketone ligands which can be compared to pentacoordinated complexes which contain only one heterocyclic *β*-diketone ligand moiety. Hence the steric crowding due to heterocyclic *β*-diketone ligand is more in hexacoordinated complexes than in pentacoordinated complexes.

To study the effect of an organic group directly attached to tin, the tin(II) complexes of compositions $\text{Sn(II)[}-\text{p}-{\text{ClC}}_{6}{\text{H}}_{4}\text{CO}\overset{⎴}{\text{C}:{\text{CON(C}}_{6}{\text{H}}_{5}\text{)N}:\text{C}}{\text{CH}}_{3}{\text{]}}_{2}$ (complex VI) and ${\text{Sn(II)[}\text{C}}_{6}{\text{H}}_{5}\text{CO}\overset{⎴}{\text{C}:{\text{CON(C}}_{6}{\text{H}}_{5}\text{)N}:\text{C}}{\text{CH}}_{3}{\text{]}}_{2}$ (complex VII) were synthesized [15]. The structure of these derivatives was proposed on the basis of spectral studies [14]. These tin(II) complexes do not possess any alkyl group on the tin atom. The tin(II) complexes were found to be least active in this series. Thus the presence of an organic group directly attached to tin is an important factor which is responsible for enhanced activity of organotin(IV) complexes.

**Figure 3:**
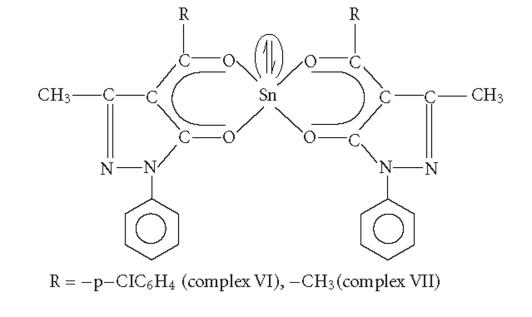
Bis(4-acyl-2,4-dihydro-5-methyl-2-phenyl-3H-pyrazol-3-onato) tin(II).

In the tin(II) complexes VI and VII, the central tin atom is surrounded by four oxygen atoms, and a lone pair of electrons is present on the tin atom (Figure 3). Complex VI was found slightly more active than complex VII. It may be due to the presence of chlorine atom in the ligand moiety of complex VI.

A detailed study of structure-activity relationship of pentacoordinated diorganotin(IV), hexacoordinated diorganotin(IV), and tetracoordinated tin(II) complexes revealed that organotin(IV) complexes are more active than tin(II) complexes containing the same organic ligand. This indicates that the alkyl group directly appended to the central tin atom is an important contributor to the activity. Further, pentacoordinated complexes in which chlorine atom is directly attached to the central tin atom (complex I, II, and III) are more active than the hexacoordinated complexes (complex IV and V) which lack chlorine atom on the central tin atom. The complexes containing −p-chloro phenyl (R = −p−Cl C_6_H_4_) group on the ligand are slightly more active than the complexes containing phenyl (R = −C_6_H_5_) group on the ligand. Tin(II)complexes (complex VI and VII) which possess a lone pair of electrons, but these complexes lack any organic group directly attached to tin, were found to be least active.

## Figures and Tables

**Table 1:** LC_50_ values of organotin(IV) and tin(II) complexes against *Musca domestica* (house fly).

No.	Complex	LC_50_ Value (in ppm)	^119^Sn NMR*

I	Me_2_SnCl[OPPMA]	14.50	−107.26
II	Me_2_SnCl[OPPMC]	15.22	−102.17
III	Me_2_SnCl[OPPMB]	16.56
IV	Me_2_Sn[OPPMA]_2_	18.16	−315.87
V	Me_2_Sn[OPPMB]_2_	20.66	−318.94
VI	Sn(II)[OPPMC]_2_	30.46
VII	Sn(II)[OPPMB]_2_	32.26

See [13].
